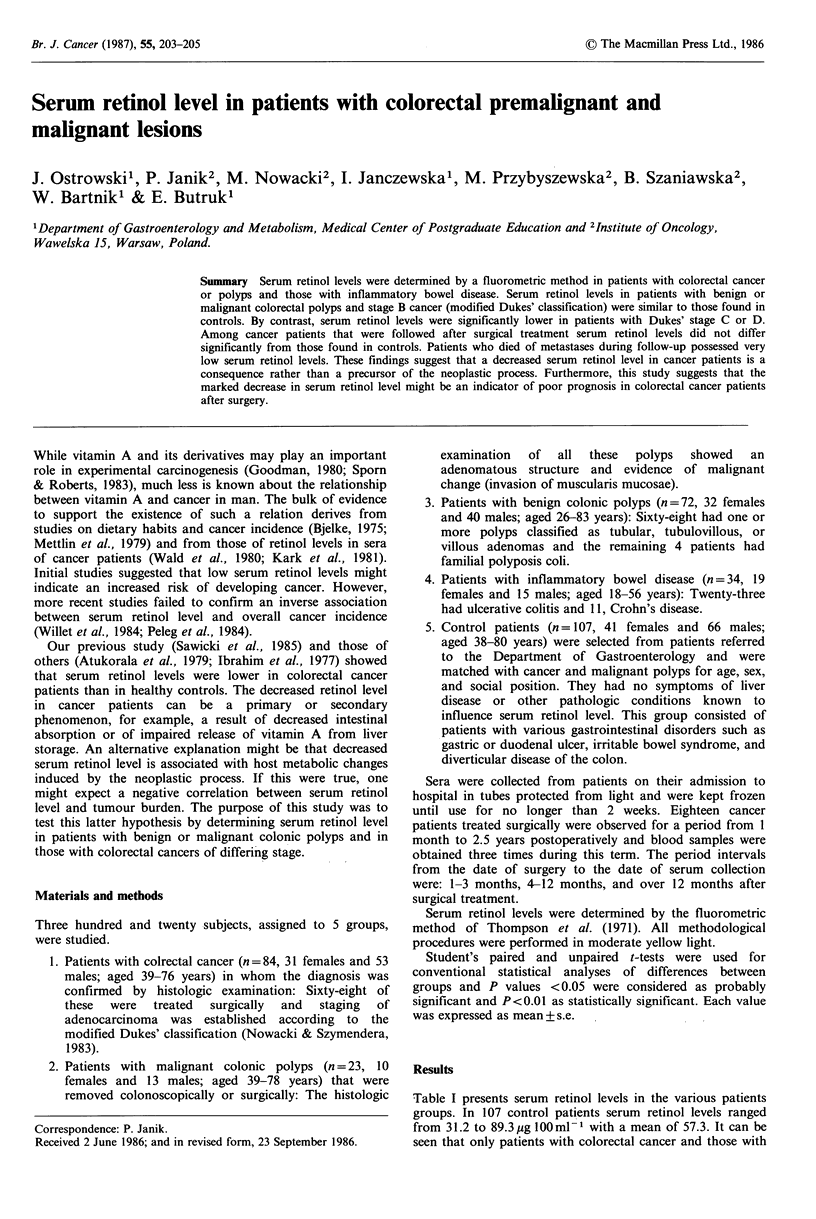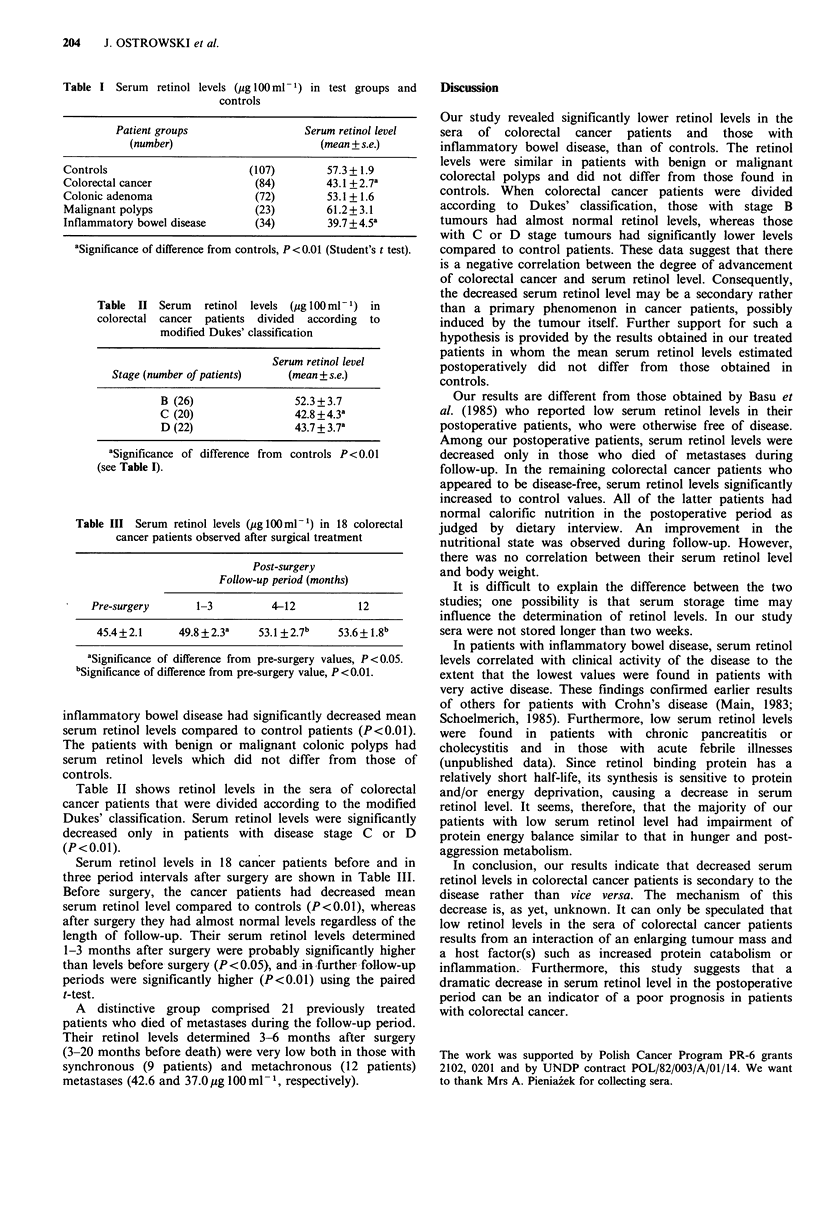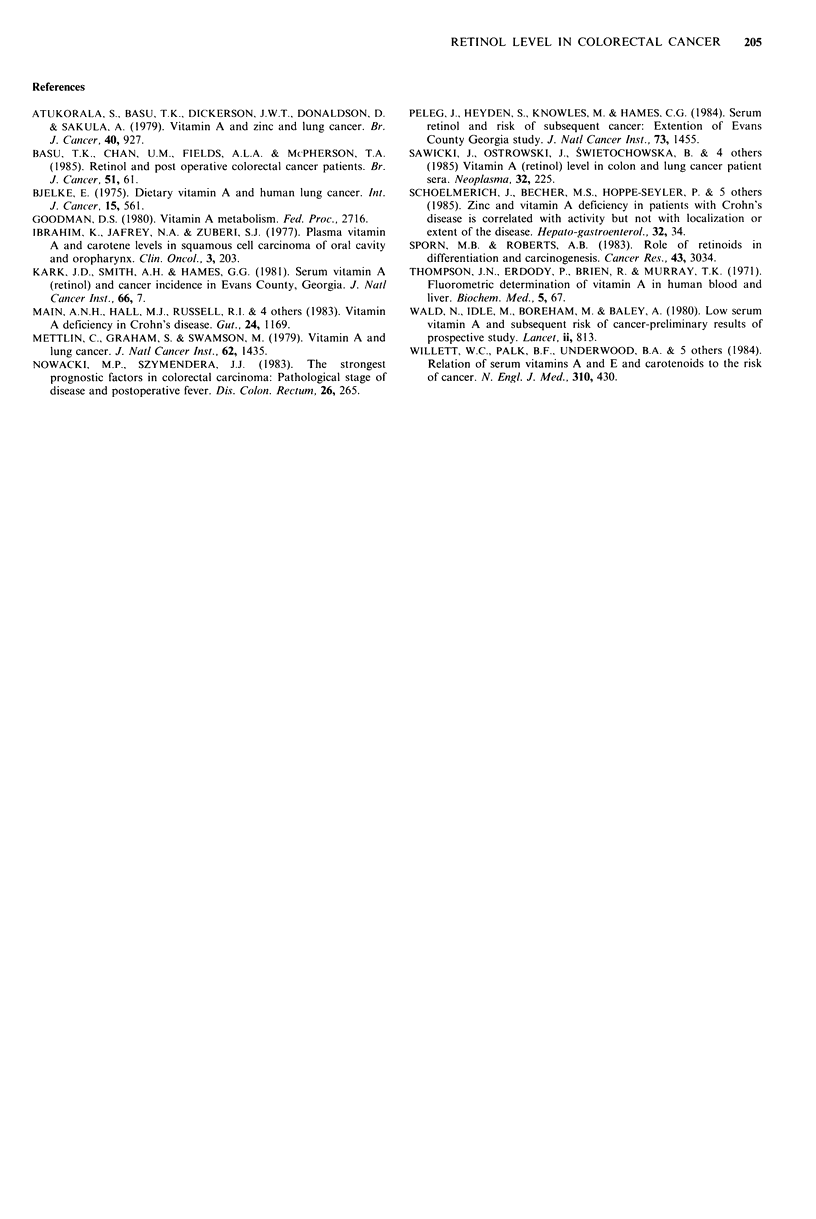# Serum retinol level in patients with colorectal premalignant and malignant lesions.

**DOI:** 10.1038/bjc.1987.38

**Published:** 1987-02

**Authors:** J. Ostrowski, P. Janik, M. Nowacki, I. Janczewska, M. Przybyszewska, B. Szaniawska, W. Bartnik, E. Butruk

## Abstract

Serum retinol levels were determined by a fluorometric method in patients with colorectal cancer or polyps and those with inflammatory bowel disease. Serum retinol levels in patients with benign or malignant colorectal polyps and stage B cancer (modified Dukes' classification) were similar to those found in controls. By contrast, serum retinol levels were significantly lower in patients with Dukes' stage C or D. Among cancer patients that were followed after surgical treatment serum retinol levels did not differ significantly from those found in controls. Patients who died of metastases during follow-up possessed very low serum retinol levels. These findings suggest that a decreased serum retinol level in cancer patients is a consequence rather than a precursor of the neoplastic process. Furthermore, this study suggests that the marked decrease in serum retinol level might be an indicator of poor prognosis in colorectal cancer patients after surgery.


*Br. J. Cancer* (1987), **55**, 203–205 © The Macmillan Press Ltd., 1986

### Serum retinol level in patients with colorectal premalignant and malignant lesions

J. Ostrowski[1], P. Janik[2], M. Nowacki[2], I. Janczewska[1], M. Przybyszewska[2], B. Szaniawska[2], W. Bartnik[1] & E. Butruk[1]

[1]*Department of Gastroenterology and Metabolism, Medical Center of Postgraduate Education and* [2]*Institute of Oncology, Wawelska 15, Warsaw, Poland.*

**Summary** Serum retinol levels were determined by a fluorometric method in patients with colorectal cancer or polyps and those with inflammatory bowel disease. Serum retinol levels in patients with benign or malignant colorectal polyps and stage B cancer (modified Dukes' classification) were similar to those found in controls. By contrast, serum retinol levels were significantly lower in patients with Dukes' stage C or D. Among cancer patients that were followed after surgical treatment serum retinol levels did not differ significantly from those found in controls. Patients who died of metastases during follow-up possessed very low serum retinol levels. These findings suggest that a decreased serum retinol level in cancer patients is a consequence rather than a precursor of the neoplastic process. Furthermore, this study suggests that the marked decrease in serum retinol level might be an indicator of poor prognosis in colorectal cancer patients after surgery.

While vitamin A and its derivatives may play an important role in experimental carcinogenesis (Goodman, 1980; Sporn & Roberts, 1983), much less is known about the relationship between vitamin A and cancer in man. The bulk of evidence to support the existence of such a relation derives from studies on dietary habits and cancer incidence (Bjelke, 1975; Mettlin *et al.*, 1979) and from those of retinol levels in sera of cancer patients (Wald *et al.*, 1980; Kark *et al.*, 1981). Initial studies suggested that low serum retinol levels might indicate an increased risk of developing cancer. However, more recent studies failed to confirm an inverse association between serum retinol level and overall cancer incidence (Willet *et al.*, 1984; Peleg *et al.*, 1984).

Our previous study (Sawicki *et al.*, 1985) and those of others (Atukorala *et al.*, 1979; Ibrahim *et al.*, 1977) showed that serum retinol levels were lower in colorectal cancer patients than in healthy controls. The decreased retinol level in cancer patients can be a primary or secondary phenomenon, for example, a result of decreased intestinal absorption or of impaired release of vitamin A from liver storage. An alternative explanation might be that decreased serum retinol level is associated with host metabolic changes induced by the neoplastic process. If this were true, one might expect a negative correlation between serum retinol level and tumour burden. The purpose of this study was to test this latter hypothesis by determining serum retinol level in patients with benign or malignant colonic polyps and in those with colorectal cancers of differing stage.

### Materials and methods

Three hundred and twenty subjects, assigned to 5 groups, were studied.

1. Patients with colrectal cancer ($n=84$, 31 females and 53 males; aged 39–76 years) in whom the diagnosis was confirmed by histologic examination: Sixty-eight of these were treated surgically and staging of adenocarcinoma was established according to the modified Dukes' classification (Nowacki & Szymendera, 1983).
2. Patients with malignant colonic polyps ($n=23$, 10 females and 13 males; aged 39–78 years) that were removed colonoscopically or surgically: The histologic examination of all these polyps showed an adenomatous structure and evidence of malignant change (invasion of muscularis mucosae).
3. Patients with benign colonic polyps ($n=72$, 32 females and 40 males; aged 26–83 years): Sixty-eight had one or more polyps classified as tubular, tubulovillous, or villous adenomas and the remaining 4 patients had familial polyposis coli.
4. Patients with inflammatory bowel disease ($n=34$, 19 females and 15 males; aged 18–56 years): Twenty-three had ulcerative colitis and 11, Crohn's disease.
5. Control patients ($n=107$, 41 females and 66 males; aged 38–80 years) were selected from patients referred to the Department of Gastroenterology and were matched with cancer and malignant polyps for age, sex, and social position. They had no symptoms of liver disease or other pathologic conditions known to influence serum retinol level. This group consisted of patients with various gastrointestinal disorders such as gastric or duodenal ulcer, irritable bowel syndrome, and diverticular disease of the colon.

Sera were collected from patients on their admission to hospital in tubes protected from light and were kept frozen until use for no longer than 2 weeks. Eighteen cancer patients treated surgically were observed for a period from 1 month to 2.5 years postoperatively and blood samples were obtained three times during this term. The period intervals from the date of surgery to the date of serum collection were: 1–3 months, 4–12 months, and over 12 months after surgical treatment.

Serum retinol levels were determined by the fluorometric method of Thompson *et al.* (1971). All methodological procedures were performed in moderate yellow light.

Student's paired and unpaired *t*-tests were used for conventional statistical analyses of differences between groups and $P$ values $<0.05$ were considered as probably significant and $P<0.01$ as statistically significant. Each value was expressed as mean$\pm$s.e.

### Results

Table I presents serum retinol levels in the various patients groups. In 107 control patients serum retinol levels ranged from 31.2 to 89.3 $\mu g\ 100\,ml^{-1}$ with a mean of 57.3. It can be seen that only patients with colorectal cancer and those with

Correspondence: P. Janik.
Received 2 June 1986; and in revised form, 23 September 1986.

**Table I** Serum retinol levels ($\mu g\,100\,ml^{-1}$) in test groups and controls

| *Patient groups (number)* | | *Serum retinol level (mean ± s.e.)* |
|---|---|---|
| Controls | (107) | 57.3±1.9 |
| Colorectal cancer | (84) | 43.1±2.7[a] |
| Colonic adenoma | (72) | 53.1±1.6 |
| Malignant polyps | (23) | 61.2±3.1 |
| Inflammatory bowel disease | (34) | 39.7±4.5[a] |

[a]Significance of difference from controls, $P<0.01$ (Student's $t$ test).

**Table II** Serum retinol levels ($\mu g\,100\,ml^{-1}$) in colorectal cancer patients divided according to modified Dukes' classification

| *Stage (number of patients)* | *Serum retinol level (mean ± s.e.)* |
|---|---|
| B (26) | 52.3±3.7 |
| C (20) | 42.8±4.3[a] |
| D (22) | 43.7±3.7[a] |

[a]Significance of difference from controls $P<0.01$ (see **Table I**).

**Table III** Serum retinol levels ($\mu g\,100\,ml^{-1}$) in 18 colorectal cancer patients observed after surgical treatment

| | *Post-surgery Follow-up period (months)* | | |
|---|---|---|---|
| *Pre-surgery* | 1–3 | 4–12 | 12 |
| 45.4±2.1 | 49.8±2.3[a] | 53.1±2.7[b] | 53.6±1.8[b] |

[a]Significance of difference from pre-surgery values, $P<0.05$.
[b]Significance of difference from pre-surgery value, $P<0.01$.

inflammatory bowel disease had significantly decreased mean serum retinol levels compared to control patients ($P<0.01$). The patients with benign or malignant colonic polyps had serum retinol levels which did not differ from those of controls.

Table II shows retinol levels in the sera of colorectal cancer patients that were divided according to the modified Dukes' classification. Serum retinol levels were significantly decreased only in patients with disease stage C or D ($P<0.01$).

Serum retinol levels in 18 cancer patients before and in three period intervals after surgery are shown in Table III. Before surgery, the cancer patients had decreased mean serum retinol level compared to controls ($P<0.01$), whereas after surgery they had almost normal levels regardless of the length of follow-up. Their serum retinol levels determined 1–3 months after surgery were probably significantly higher than levels before surgery ($P<0.05$), and in further follow-up periods were significantly higher ($P<0.01$) using the paired $t$-test.

A distinctive group comprised 21 previously treated patients who died of metastases during the follow-up period. Their retinol levels determined 3–6 months after surgery (3–20 months before death) were very low both in those with synchronous (9 patients) and metachronous (12 patients) metastases (42.6 and 37.0 $\mu g\,100\,ml^{-1}$, respectively).

### Discussion

Our study revealed significantly lower retinol levels in the sera of colorectal cancer patients and those with inflammatory bowel disease, than of controls. The retinol levels were similar in patients with benign or malignant colorectal polyps and did not differ from those found in controls. When colorectal cancer patients were divided according to Dukes' classification, those with stage B tumours had almost normal retinol levels, whereas those with C or D stage tumours had significantly lower levels compared to control patients. These data suggest that there is a negative correlation between the degree of advancement of colorectal cancer and serum retinol level. Consequently, the decreased serum retinol level may be a secondary rather than a primary phenomenon in cancer patients, possibly induced by the tumour itself. Further support for such a hypothesis is provided by the results obtained in our treated patients in whom the mean serum retinol levels estimated postoperatively did not differ from those obtained in controls.

Our results are different from those obtained by Basu *et al.* (1985) who reported low serum retinol levels in their postoperative patients, who were otherwise free of disease. Among our postoperative patients, serum retinol levels were decreased only in those who died of metastases during follow-up. In the remaining colorectal cancer patients who appeared to be disease-free, serum retinol levels significantly increased to control values. All of the latter patients had normal calorific nutrition in the postoperative period as judged by dietary interview. An improvement in the nutritional state was observed during follow-up. However, there was no correlation between their serum retinol level and body weight.

It is difficult to explain the difference between the two studies; one possibility is that serum storage time may influence the determination of retinol levels. In our study sera were not stored longer than two weeks.

In patients with inflammatory bowel disease, serum retinol levels correlated with clinical activity of the disease to the extent that the lowest values were found in patients with very active disease. These findings confirmed earlier results of others for patients with Crohn's disease (Main, 1983; Schoelmerich, 1985). Furthermore, low serum retinol levels were found in patients with chronic pancreatitis or cholecystitis and in those with acute febrile illnesses (unpublished data). Since retinol binding protein has a relatively short half-life, its synthesis is sensitive to protein and/or energy deprivation, causing a decrease in serum retinol level. It seems, therefore, that the majority of our patients with low serum retinol level had impairment of protein energy balance similar to that in hunger and post-aggression metabolism.

In conclusion, our results indicate that decreased serum retinol levels in colorectal cancer patients is secondary to the disease rather than *vice versa*. The mechanism of this decrease is, as yet, unknown. It can only be speculated that low retinol levels in the sera of colorectal cancer patients results from an interaction of an enlarging tumour mass and a host factor(s) such as increased protein catabolism or inflammation. Furthermore, this study suggests that a dramatic decrease in serum retinol level in the postoperative period can be an indicator of a poor prognosis in patients with colorectal cancer.

The work was supported by Polish Cancer Program PR-6 grants 2102, 0201 and by UNDP contract POL/82/003/A/01/14. We want to thank Mrs A. Pieniażek for collecting sera.

### References

ATUKORALA, S., BASU, T.K., DICKERSON, J.W.T., DONALDSON, D. & SAKULA, A. (1979). Vitamin A and zinc and lung cancer. *Br. J. Cancer*, **40,** 927.

BASU, T.K., CHAN, U.M., FIELDS, A.L.A. & McPHERSON, T.A. (1985). Retinol and post operative colorectal cancer patients. *Br. J. Cancer*, **51,** 61.

BJELKE, E. (1975). Dietary vitamin A and human lung cancer. *Int. J. Cancer*, **15,** 561.

GOODMAN, D.S. (1980). Vitamin A metabolism. *Fed. Proc.*, 2716.

IBRAHIM, K., JAFREY, N.A. & ZUBERI, S.J. (1977). Plasma vitamin A and carotene levels in squamous cell carcinoma of oral cavity and oropharynx. *Clin. Oncol.*, **3,** 203.

KARK, J.D., SMITH, A.H. & HAMES, G.G. (1981). Serum vitamin A (retinol) and cancer incidence in Evans County, Georgia. *J. Natl Cancer Inst.*, **66,** 7.

MAIN, A.N.H., HALL, M.J., RUSSELL, R.I. & 4 others (1983). Vitamin A deficiency in Crohn's disease. *Gut.*, **24,** 1169.

METTLIN, C., GRAHAM, S. & SWAMSON, M. (1979). Vitamin A and lung cancer. *J. Natl Cancer Inst.*, **62,** 1435.

NOWACKI, M.P., SZYMENDERA, J.J. (1983). The strongest prognostic factors in colorectal carcinoma: Pathological stage of disease and postoperative fever. *Dis. Colon. Rectum*, **26,** 265.

PELEG, J., HEYDEN, S., KNOWLES, M. & HAMES, C.G. (1984). Serum retinol and risk of subsequent cancer: Extention of Evans County Georgia study. *J. Natl Cancer Inst.*, **73,** 1455.

SAWICKI, J., OSTROWSKI, J., ŚWIETOCHOWSKA, B. & 4 others (1985) Vitamin A (retinol) level in colon and lung cancer patient sera. *Neoplasma*, **32,** 225.

SCHOELMERICH, J., BECHER, M.S., HOPPE-SEYLER, P. & 5 others (1985). Zinc and vitamin A deficiency in patients with Crohn's disease is correlated with activity but not with localization or extent of the disease. *Hepato-gastroenterol.*, **32,** 34.

SPORN, M.B. & ROBERTS, A.B. (1983). Role of retinoids in differentiation and carcinogenesis. *Cancer Res.*, **43,** 3034.

THOMPSON, J.N., ERDODY, P., BRIEN, R. & MURRAY, T.K. (1971). Fluorometric determination of vitamin A in human blood and liver. *Biochem. Med.*, **5,** 67.

WALD, N., IDLE, M., BOREHAM, M. & BALEY, A. (1980). Low serum vitamin A and subsequent risk of cancer-preliminary results of prospective study. *Lancet*, **ii,** 813.

WILLETT, W.C., PALK, B.F., UNDERWOOD, B.A. & 5 others (1984). Relation of serum vitamins A and E and carotenoids to the risk of cancer. *N. Engl. J. Med.*, **310,** 430.